# Downregulation of *GSTK1* Is a Common Mechanism Underlying Hypertrophic Cardiomyopathy

**DOI:** 10.3389/fphar.2016.00162

**Published:** 2016-06-14

**Authors:** Shota Sasagawa, Yuhei Nishimura, Shiko Okabe, Soichiro Murakami, Yoshifumi Ashikawa, Mizuki Yuge, Koki Kawaguchi, Reiko Kawase, Ryuji Okamoto, Masaaki Ito, Toshio Tanaka

**Affiliations:** ^1^Department of Systems Pharmacology, Mie University Graduate School of Medicine, TsuJapan; ^2^Department of Molecular and Cellular Pharmacology, Pharmacogenomics and Pharmacoinformatics, Mie University Graduate School of Medicine, TsuJapan; ^3^Mie University Medical Zebrafish Research Center, TsuJapan; ^4^Department of Omics Medicine, Mie University Industrial Technology Innovation Institute, TsuJapan; ^5^Department of Bioinformatics, Mie University Life Science Research Center, TsuJapan; ^6^Department of Cardiology and Nephrology, Mie University Graduate School of Medicine, TsuJapan

**Keywords:** hypertrophic cardiomyopathy, comparative transcriptomics, GSTK1, mitochondria, oxidative stress, zebrafish, CRISPR/Cas9, systems pharmacology

## Abstract

Hypertrophic cardiomyopathy (HCM) is characterized by left ventricular hypertrophy and is associated with a number of potential outcomes, including impaired diastolic function, heart failure, and sudden cardiac death. Various etiologies have been described for HCM, including pressure overload and mutations in sarcomeric and non-sarcomeric genes. However, the molecular pathogenesis of HCM remains incompletely understood. In this study, we performed comparative transcriptome analysis to identify dysregulated genes common to five mouse HCM models of differing etiology: (i) mutation of myosin heavy chain 6, (ii) mutation of tropomyosin 1, (iii) expressing human phospholamban on a null background, (iv) knockout of frataxin, and (v) transverse aortic constriction. Gene-by-gene comparison identified five genes dysregulated in all five HCM models. Glutathione S-transferase kappa 1 (*Gstk1*) was significantly downregulated in the five models, whereas myosin heavy chain 7 (*Myh7*), connective tissue growth factor (*Ctgf*), periostin (*Postn*), and reticulon 4 (*Rtn4)* were significantly upregulated. Gene ontology comparison revealed that 51 cellular processes were significantly enriched in genes dysregulated in each transcriptome dataset. Among them, six processes (oxidative stress, aging, contraction, developmental process, cell differentiation, and cell proliferation) were related to four of the five genes dysregulated in all HCM models. *GSTK1* was related to oxidative stress only, whereas the other four genes were related to all six cell processes except *MYH7* for oxidative stress. Gene–gene functional interaction network analysis suggested correlative expression of *GSTK1*, *MYH7*, and actin alpha 2 (*ACTA2*). To investigate the implications of Gstk1 downregulation for cardiac function, we knocked out *gstk1* in zebrafish using the clustered regularly interspaced short palindromic repeats/Cas9 system. We found that expression of the zebrafish homologs of *MYH7*, *ACTA2*, and actin alpha 1 were increased in the *gstk1*-knockout zebrafish. *In vivo* imaging of zebrafish expressing a fluorescent protein in cardiomyocytes showed that *gstk1* deletion significantly decreased the end diastolic volume and, to a lesser extent, end systolic volume. These results suggest that downregulation of *GSTK1* may be a common mechanism underlying HCM of various etiologies, possibly through increasing oxidative stress and the expression of sarcomere genes.

## Introduction

Hypertrophic cardiomyopathy is characterized by thickening of the left ventricle and is associated with a range of potential outcomes, such as impaired diastolic function, heart failure, and sudden cardiac death (Semsarian et al., 2015). The prevalence of HCM is estimated to be ~1 in 500 people (Semsarian et al., 2015). HCM has multiple etiologies, including mutation in sarcomeric genes such as myosin heavy chain 7 (*MYH7*) and tropomyosin 1 (*TPM1*) and in non-sarcomeric genes such as *PLN* and *FXN* (Ho et al., 2015). HCM is also caused by pressure overload (Lai et al., 2014; Aubert et al., 2016). However, the molecular mechanisms underlying HCM remain incompletely understood (Force et al., 2010). In general, mutations in *MYH7* and other myosin genes associated with HCM increase the force-generating capacity of the sarcomere rather than diminish its function (Poggesi and Ho, 2014). In addition, most HCM-associated mutations in thin filament regulatory proteins such as *TPM1* increase the Ca^2++^ sensitivity of force production (Ashrafian et al., 2011). These findings suggest that compensatory hypertrophy is unlikely to be the cause of HCM induced by mutation of sarcomeric genes (Ashrafian et al., 2011). PLN regulates sarcoplasmic reticulum Ca^2+^ cycling in the heart through inhibition of ATPase sarcoplasmic/endoplasmic reticulum Ca^2+^ transporting 2 (ATP2A2) (Wang et al., 2011). Mutation of *PLN* causing superinhibition of ATP2A2 can cause HCM (Wang et al., 2011). Haploinsufficiency of *ATP2A2* can also cause HCM, possibly through mitochondrial dysfunction (Prasad et al., 2015), suggesting that mutation of *PLN* causing HCM may impair mitochondrial function. Haploinsufficiency of *FXN* is a major cause of FA (Payne and Wagner, 2012). FA is associated with progressive HCM, and this is a common cause of death in FA patients (Payne and Wagner, 2012). FXN is an iron-binding protein targeted to the mitochondrial matrix, and consistent with this, mitochondrial function is impaired in FA (Payne and Wagner, 2012). Mitochondrial dysfunction has also been detected in HCM caused by mutation in sarcomeric genes (Lucas et al., 2003) and pressure overload (Doenst et al., 2013). These findings suggest the existence of convergent pathways that cause HCM by impairment of mitochondrial function.

Comparative transcriptomics could represent a new frontier in the search for novel biomarkers and/or therapeutic targets in diseases with multiple etiologies because it facilitates the identification of dysregulated genes common to all disease etiologies (Sasagawa et al., 2016b). In this study, we sought to identify DEGs common to five different mouse models of HCM. The transcriptome datasets were downloaded from a public database (Barrett et al., 2009) and were derived from mouse models of HCM caused by: (i) mutation of myosin heavy chain 6 (*Myh6*) (Luczak et al., 2011), (ii) mutation of *Tpm1* (Rajan et al., 2013), (iii) expressing human PLN on a null background (Wang et al., 2011), (iv) KO of *Fxn* (Huang et al., 2013), and (v) TAC, a model of pressure overload-induced HCM (Lai et al., 2014). We identified five genes dysregulated in all five HCM transcriptome datasets, among which glutathione S-transferase kappa 1 (*Gstk1*) was the only gene downregulated. We were particularly interested in this gene because Gstk1 is localized in mitochondria and peroxisomes (Petit et al., 2009). We examined the function of gstk1 in zebrafish, which has emerged as a useful *in vivo* model to study human genetic disorders including HCM (Becker et al., 2012). We demonstrate here that knockout of *gstk1* in zebrafish increased the expression of HCM marker genes and decreased the cardiac EDV and, to a lesser extent, the ESV, suggesting that downregulation of *GSTK1* may be a common mechanism underlying HCM of various etiologies.

## Materials and Methods

### Ethics Statement

This study was carried out in strict accordance with Japanese law [The Humane Treatment and Management of Animals (2014), Standards Relating to the Care and Management of Laboratory Animals and Relief of Pain (2013), and the Guidelines for Proper Conduct of Animal Experiments (Science Council of Japan, 2006)] (Science Council of Japan, 2006; Ministry of the Environment Japan, 2013, 2014). All efforts were made to minimize animal suffering. Mie University Institutional Animal Care and Use Committee guidelines state that no approval is required for experiments using zebrafish.

### Comparative Transcriptome Analysis

Among the transcriptome datasets analyzing HCM caused by mutation or KO of 23 genes listed in Table 1 of Ho et al. (2015), including cardiac troponin T (*TNNT2*), cardiac troponin I (*TNNI3*), and cardiac myosin-binding protein C (*MYBPC3*), in the GEO (Barrett et al., 2009), we selected datasets satisfying all of the following criteria: (i) using mice 2–4 months old when RNA was extracted, (ii) using mice fed with a standard diet without any supplementation, (iii) analyzing the expression profile of mRNAs but not microRNAs or non-coding RNAs, (iv) with downloadable raw data or normalized data, with the quality of each probe signal available, and (v) having control and HCM groups with at least two samples in each group. Four transcriptome datasets passed these criteria. In the Myh6 model (GSE25700) (Luczak et al., 2011), C57BL/6 mice overexpressed a rat *Myh6* transgene containing a point mutation (R403Q) and a deletion of amino acids 468–527 was replaced with nine non-myosin amino acids (Myh6-R403Q-d50) (Vikstrom et al., 1996). Hearts from wild-type or HCM C57BL/6 mice were excised at 2 months of age and subjected to transcriptome analysis. In the Tpm1 mutation model (GSE42892; Rajan et al., 2013), FVB/N mice overexpressed mouse *Tpm1* containing a point mutation resulting in E180G (Tpm1-E180G). Hearts from wild-type or HCM FVB/N mice were excised at 4 months of age and subjected to transcriptome analysis. In the human PLN model (GSE20172) (Wang et al., 2011), hearts from wild-type or transgenic mice expressing human PLN on a null background were excised at 11 weeks of age and used for transcriptome analysis. In the Fxn-KO model (GSE31208; Huang et al., 2013), hearts from wild-type or muscle creatine kinase conditional Fxn-KO mice were excised at 10 weeks of age and subjected to transcriptome analysis. We also included a transcriptome dataset analyzing HCM caused by TAC, because it is a representative murine model of cardiac hypertrophy (Rockman et al., 1991). In the TAC model (GSE56348; Lai et al., 2014), hearts were excised from C57BL/6J mice 1 month after TAC or sham surgery performed at 8-week-old and subjected to transcriptome analysis. The raw data were normalized using “affy” (Gautier et al., 2004) for GSE25700 and GSE31208, “oligo” (Carvalho et al., 2007) for GSE42892 and GSE56348, and “limma” (Ritchie et al., 2015) for GSE20172 in Bioconductor (Gentleman et al., 2004). Probes with reliable signals were selected and subjected to “RankProd” (Hong et al., 2006) to identify DEGs in the HCM mice compared with the relevant control mice using a false discovery rate (FDR) of 20% as the threshold. The gene symbols of the DEGs in each model were converted to those of the human orthologous genes using Life Science Knowledge Bank (World Fusion, Tokyo, Japan). The list of DEGs in each HCM model is shown in Supplementary Tables S1-1–S1-5.

### Bioinformatic Analysis of the DEGs in the Five HCM Models

To identify cellular processes significantly enriched for each DEG identified in each HCM model, we used Pathway Studio (Nikitin et al., 2003) that uses gene sets derived from natural language processing-based text mining of published literature in relation to biological functions such as cellular processes, expression targets, and binding partners. The lists of DEGs shown in Supplementary Tables S1-1–S1-5 were subjected to Pathway Studio and used to predict the cellular processes significantly enriched, using subnetwork enrichment analysis. The predicted cellular processes with *p* < 1.0 × 10^-5^ are shown in Supplementary Tables S2-1–S2-5. Common cellular processes among Supplementary Tables S2-1–S2-5 are shown in Supplementary Table S2-6.

To identify networks related to the five genes dysregulated in all five mouse HCM models, we used GeneMANIA in Cytoscape (Shannon et al., 2003) with the default settings. GeneMANIA uses a database of organism-specific weighted networks to construct a weighted composite functional interaction network between a pair of genes, including physical interaction, co-expression, pathway, co-localization, and shared protein domains, from a list of genes (Montojo et al., 2014). The networks related to the five common DEGs are shown in Supplementary Tables S3-1–S3-6. The gene score calculated based on these functional interactions is shown in Supplementary Table S3-7.

We used iRegulon to identify TFs potentially regulating the five DEGs common to the HCM models (Janky et al., 2014). iRegulon exploits the fact that genes co-regulated by the same TF often contain common TF-binding sites. iRegulon has been successfully used to identify TFs in gene lists (Sasagawa et al., 2016a) using ENCODE ChIP-seq data as a reference database (Gerstein et al., 2012). The five common DEGs were subjected to iRegulon using a normalized enrichment score of 5 as the threshold. The predicted TFs are listed in Supplementary Table S4.

### Zebrafish Strains

We obtained Tg (myl7:mRFP) zebrafish, which express mRFP under the control of the myosin light chain 7 (*myl7*) promoter, a gene selectively expressed in cardiomyocytes (Kawahara et al., 2009), from the National BioResource Project Zebrafish (Saitama, Japan). Zebrafish were bred and maintained according to previously described methods (Westerfield, 2007; Nishimura et al., 2016). Briefly, zebrafish were raised at 28.5°C ± 0.5°C with a 14-h/10-h light/dark cycle. Embryos were obtained by natural mating and cultured in 0.3× Danieau’s solution (19.3 mM NaCl, 0.23 mM KCl, 0.13 mM MgSO_4_, 0.2 mM Ca(NO_3_)_2_, 1.7 mM HEPES, pH 7.2) until 5 dpf, at which time they were used for the *in vivo* imaging analyses or were processed for qPCR.

### Knockout of *gstk1* in Zebrafish

KO of *gstk1* in zebrafish was performed by the ready-to-use CRISPR/Cas9 method (Aida et al., 2015). CRISPR RNA (crRNA) targeting a 5′-ATTGTCTCAAAAACGTTGGA-3′ sequence in the *gstk1* genome and trans-activating crRNA (tracrRNA) were obtained from FASMAC (Kanagawa, Japan). Recombinant Cas9 protein was obtained from Toolgen (Seoul, South Korea). In brief, crRNA, tracrRNA, and Cas9 protein were dissolved in sterilized water at concentrations of 250, 1000, and 1000 ng/μL, respectively, and stored at -80°C until required. For microinjection, the crRNA, tracrRNA, Cas9 protein, and a lissamine-labeled control morpholino with no known target gene (Gene Tools, Philomath, OR, USA) were mixed in Yamamoto’s Ringer’s solution (0.75% NaCl, 0.02% KCl, 0.02% CaCl_2_, 0.002% NaHCO_3_) to final concentrations of 100, 100, 400 ng/μL, and 50 nM, respectively. The solution was injected into one- to four-cell-stage zebrafish embryos derived from the Tg (myl7:mRFP) line.

At 1 dpf, the embryos exhibiting bright lissamine fluorescence were selected and maintained until 5 dpf. At 5 dpf, the selected zebrafish were used for *in vivo* imaging of the cardiac ventricles or were processed for qPCR. After completion of the *in vivo* imaging experiments, genomic DNA was extracted from the zebrafish by incubation in 50 μL of lysis buffer (10 mM Tris–HCl, pH 8.0, 0.1 mM EDTA, 0.2% Triton X-100, 200 μg/mL proteinase K) at 55°C overnight, followed by incubation at 99°C for 10 min. The solution was then placed at 4°C and used as the template for PCR. To detect the crRNA-induced mutations, we performed a heteroduplex mobility assay (Kotani et al., 2015). Briefly, a short fragment of the *gstk1* gene encompassing the crRNA target sites was amplified from the genomic DNA using gstk1_gF1 and gstk1_gR1 primers and QuickTaq (Toyobo, Osaka, Japan). PCR cycling conditions were: 94°C for 2 min followed by 40 cycles of 94°C for 30 s, 60°C for 30 s, and 68°C for 30 s. The PCR products were electrophoresed on 10% polyacrylamide gels (Wako Chemicals) and visualized by ethidium bromide staining. The result is shown in Supplementary Figure S2. The crRNA, tracrRNA, and PCR primer sequences are shown in Supplementary Table S5.

### *In Vivo* Imaging of the Zebrafish Heart

Tg (myl7:mRFP) zebrafish at 5 dpf were transferred onto glass slides. A few drops of 3% low-melting point agarose were laid over the living larvae, which were immediately placed on their backs. The ventricles of the embedded larvae were observed using an epifluorescence microscope (SMZ25; Nikon, Tokyo, Japan) with RFP filters, and images were recorded at 100 frames/s for 10 s. Quantitative assessment of cardiac function was performed using ImageJ (Schneider et al., 2012) and Volocity software (Perkin Elmer, Cambridge, MA, USA). Briefly, the time-lapse images were processed using the Fast Fourier Transform package in ImageJ to reduce the background noise, and the long and short diastolic and systolic diameters of the ventricle were measured using Volocity. The EDV and ESV were calculated using the diameters. The EF was calculated from the EDV and ESV.

### Quantitative PCR Analysis

Total RNA was extracted from control or *gstk1*-KO zebrafish at 5 dpf using an RNAqueous Micro Kit (Takara, Kyoto, Japan) according to the manufacturer’s protocol. RNA concentrations were determined using a NanoDrop spectrophotometer (Thermo Scientific, Waltham, MA, USA), and cDNAs were generated using a ReverTra Ace qPCR RT Kit (Toyobo). Quantitative PCR (qPCR) was performed using an ABI Prism 7300 (Life Technologies Carlsbad, CA, USA) with THUNDERBIRD SYBR qPCR Mix (Toyobo). The thermal cycling conditions were: 95°C for 1 min followed by 40 cycles of 95°C for 15 s, 60°C for 15 s, and 72°C for 45 s. We measured the expression of actin alpha 1a (*acta1a*), actin alpha 1b (*acta1b*), ventricular myosin heavy chain (*vmhc*), actin-related protein 1 (*arp1*), connective tissue growth factor a (*ctgfa*), periostin, osteoblast specific factor b (*postnb*) and glyceraldehyde-3-phosphate dehydrogenase (*gapdh*) mRNA. The *acta1a*, *acta1b*, *vmhc*, *arp1*, *ctgfa*, and *postnb* mRNA levels were normalized to *gapdh* mRNA levels to correct for variability in the initial template concentration and the conversion efficiency of the reverse transcription reaction. The primer sequences are shown in Supplementary Table S5.

### Statistical Analysis

Statistical analysis was performed using Prism 6 (GraphPad, La Jolla, CA, USA). Group means were compared by the Mann–Whitney *U* test with alpha set at 0.05. Data are shown as the mean ± standard error (SEM).

## Results

### Identification of Dysregulated Genes Common to the Five Mouse HCM Models

**FIGURE 1:**
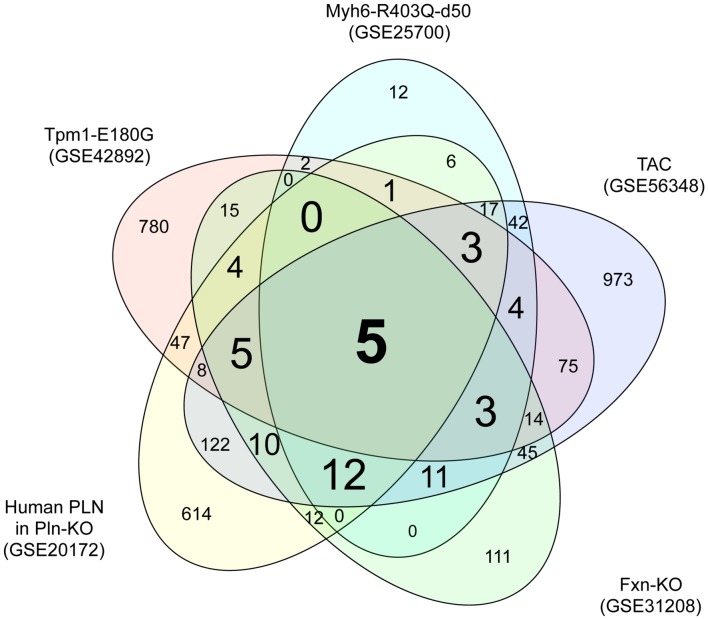
**Venn diagram of differentially expressed genes in the five hypertrophic cardiomyopathy (HCM) transcriptome datasets.** Transcriptome data from the mouse HCM models caused by (i) mutation of Myh6 (GSE25700), (ii) mutation of Tpm1 (GSE42892), (iii) human PLN transgene (GSE20172), (iv) KO of Fxn (GSE31208), and (v) TAC (GSE56348) were downloaded from GEO. Genes that were differentially expressed in the HCM and control groups of each dataset were identified using a FDR of 20% as the threshold. The numbers of differentially expressed genes unique to each transcriptome dataset and shared between datasets are shown.

To identify genes dysregulated in HCM of differing etiologies, we downloaded transcriptome datasets from studies of five mouse models of HCM (Luczak et al., 2011; Wang et al., 2011; Huang et al., 2013; Rajan et al., 2013; Lai et al., 2014) from GEO (Barrett et al., 2009). We identified 966, 118, 866, 247, and 1349 DEGs in HCM caused by mutation of *Myh6*, mutation of *Tpm1*, a human PLN transgene, KO of *Fxn1*, and TAC, respectively, compared with the relevant controls (Supplementary Tables S1-1–S1-5). A Venn diagram showing unique and shared DEGs is shown in **Figure 1**. Five DEGs were either upregulated or downregulated in all five datasets (**Table 1**). Expression of glutathione S-transferase kappa 1 (*Gstk1*) was significantly decreased in the five HCM transcriptome datasets, whereas connective tissue growth factor (*Ctgf*), myosin heavy chain 7 (*Myh7*), periostin (*Postn*), and reticulon 4 (*Rtn4*) were significantly increased in all datasets. These results suggest that these five DEGs may be robust biomarkers of HCM caused by multiple mechanisms.

**Table 1:** Differentially expressed genes common to the five hypertrophic cardiomyopathy (HCM) transcriptome datasets.

Mm symbol	Hs symbol	Gene name	Myh6-R403Q-d50 HCM: *N* = 8, C: *N* = 8 (GSE25700)		Tpm1-E180G HCM: *N* = 3, C: *N* = 3 (GSE42892)		human PLN HCM: *N* = 6, C: *N* = 6 (GSE20172)		Fxn-KO HCM: *N* = 2, C: *N* = 2 (GSE31208)		TAC HCM: *N* = 5, C: *N* = 5 (GSE56348)	
			log (HCM/C)	FDR	log (HCM/C)	FDR	log (HCM/C)	FDR	log (HCM/C)	FDR	log (HCM/C)	FDR
*Gstkl*	*GSTK1*	Glutathione S-transferase kappa 1	-0.58	0.00	-0.92	0.15	-0.36	0.20	-2.93	0.01	-0.54	0.03
*Ctgf*	*CTGF*	Connective tissue growth factor	1.62	0.00	1.06	0.06	1.03	0.00	2.43	0.02	1.30	0.00
*Myh7*	*MYH7*	myosin heavy chain 7	1.36	0.00	1.87	0.00	1.11	0.00	3.06	0.01	2.29	0.00
*Postn*	*POSTN*	pre host in	0.76	0.00	1.86	0.00	0.48	0.11	3.67	0.00	2.39	0.00
*Rtn4*	*RTN4*	Reticulon 4	0.60	0.03	0.84	0.13	0.64	0.01	1.26	0.20	0.65	0.00

### Identification of Functional Interaction Networks Related to the Common DEGs

**FIGURE 2:**
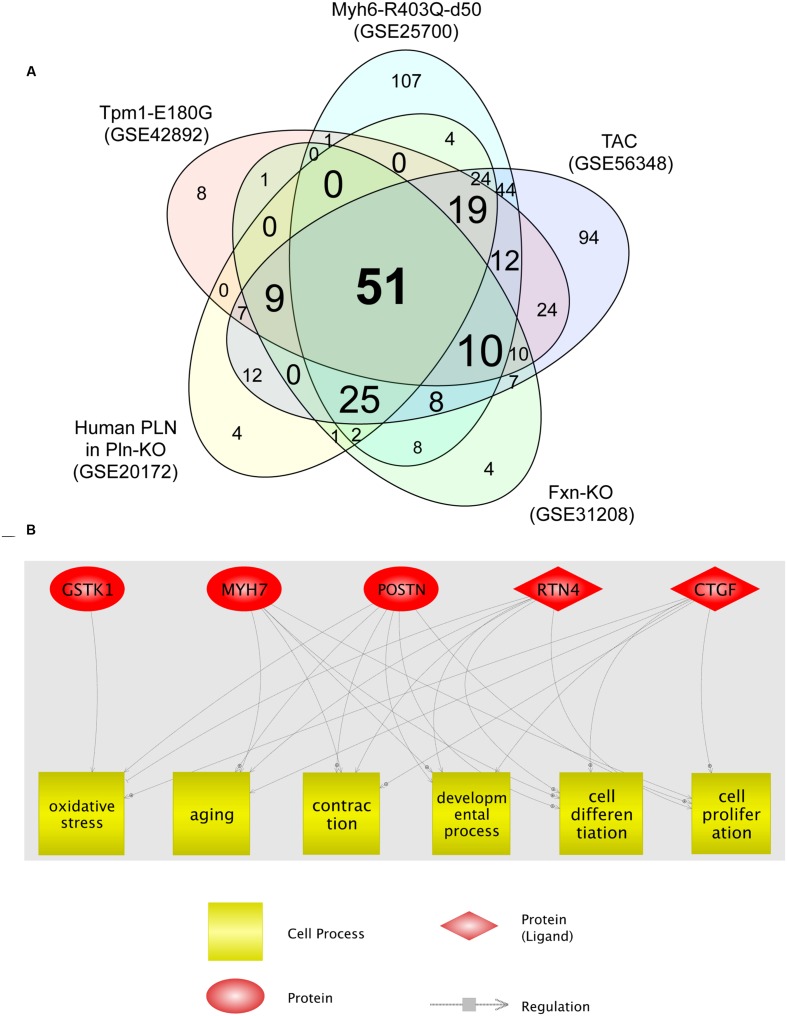
**Cell processes related to the five genes dysregulated in all five HCM transcriptome datasets. (A)** Venn diagram of cellular processes significantly enriched for differentially expressed genes identified in each HCM transcriptome dataset. The numbers of cellular processes unique to each transcriptome dataset and shared between datasets are shown. **(B)** Network among the five genes dysregulated in all five HCM transcriptome datasets, and cellular processes that connect four of the five dysregulated genes.

We then analyzed the cellular processes significantly enriched for the DEGs identified in each HCM transcriptome dataset. The identified processes are shown in Supplementary Tables S2-1–S2-5. Fifty-one cellular processes were common to the five transcriptome datasets (**Figure 2A**; Supplementary Table S2-6). Among these, six (oxidative stress, aging, contraction, developmental process, cell differentiation, and cell proliferation) were related to four of the five genes dysregulated in all HCM models (**Figure 2B**). *GSTK1* was related to oxidative stress only, whereas the other four genes were related to all six cell processes, except *MYH7* for oxidative stress. These results suggest that increased oxidative stress may be a common pathophysiological mechanism in HCM.

**FIGURE 3:**
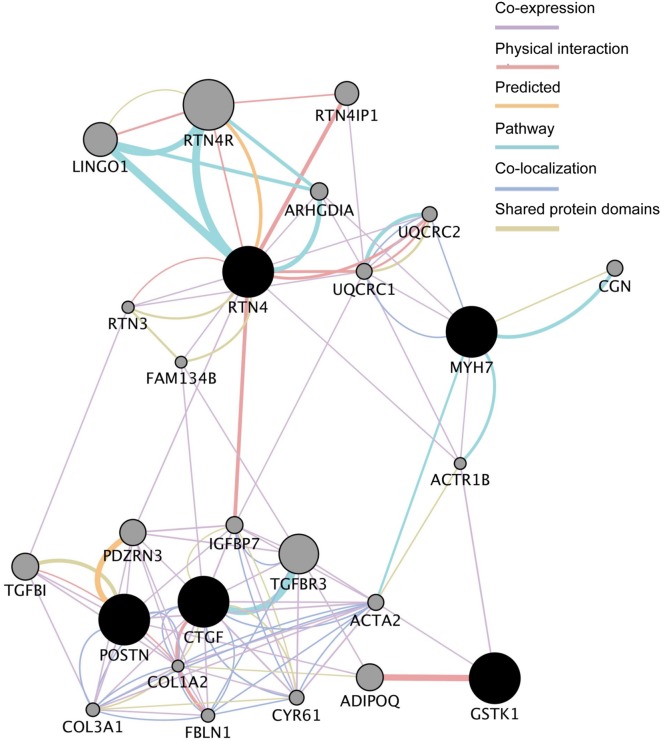
**Functional interaction networks related to the five genes dysregulated in all five HCM transcriptome datasets.** The five genes (shown in black circles) were subjected to GeneMANIA searches. The functional interaction networks identified by GeneMANIA are shown. Co-expression (Supplementary Table S3-1), physical interaction (Supplementary Table S3-2), predicted (Supplementary Table S3-3), pathway (Supplementary Table S3-4), co-localization (Supplementary Table S3-5), and shared protein domain (Supplementary Table S3-6) interactions are shown in purple, russet, orange, sky, aqua, and green lines, respectively. The size of the gray circles denotes the score in the functional network (Supplementary Table S3-7).

We next analyzed the functional interaction networks related to the five common DEGs using GeneMANIA (Montojo et al., 2014). The networks identified by GeneMANIA are shown in **Figure 3** and Supplementary Tables S3-1–S3-7. *GSTK1* is connected to *MYH7* and *RTN4* through actin-related protein 1 homolog B (*ACTR1B*) and to *CTGF* and *POSTN* through actin α2 (*ACTA2*) as co-expressed genes (Supplementary Table S3-1). GSTK1 is also connected to adiponectin (ADIPOQ) by sharing a physical interaction (Supplementary Table S3-2). These results suggest that down-regulation of *GSTK1* may affect the expression of co-expressed genes in the network and impair pathways related to ADIPOQ.

We also analyzed the TFs potentially regulating the common DEGs using iRegulon (Janky et al., 2014). The identified TFs are shown in Supplementary Figure S1 and Table S4. Eight TFs were identified, including TATA-binding protein (TBP), SRF, TEA domain family member 1 (TEAD1), and nuclear transcription factor Y beta (NFYB) as TFs possibly regulating the transcription of *MYH7*, *CTGF*, *RTN4*, and/or *POSTN*. SRF is known to be activated in HCM (Chai and Tarnawski, 2002). These results suggest that SRF may be activated in the five HCM models. However, no TFs were identified in this analysis as regulating GSTK1 (Supplementary Table S4).

### gstk1 Knockout Increases Expression of the Zebrafish Homologs of *ACTA2*

**FIGURE 4:**
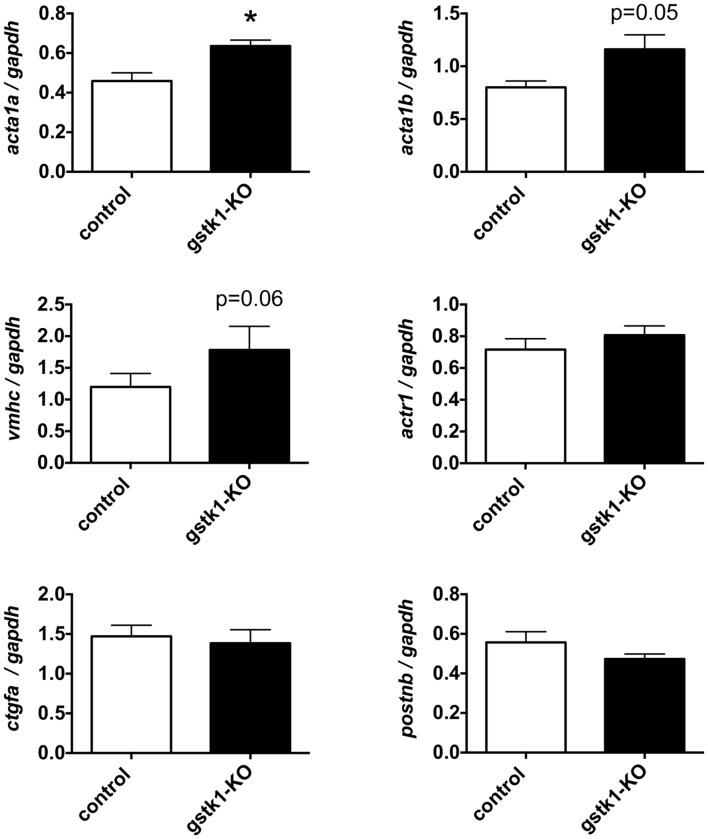
***gstk1* knockout in zebrafish increases the expression of *acta1a* qPCR analysis of *acta1a*, *acta1b*, *vmhc*, *actr1*, *ctgfa*, and *postnb* mRNA levels in control and *gstk1*-knockout (KO) zebrafish at 5 dpf.** Expression was normalized to *gapdh* mRNA levels. *N* = 8 per group. *p < 0.05 vs. control group.

To determine whether reduction of GSTK1 affects expression of genes co-expressed in the functional interaction networks identified by GeneMANIA, we knocked out *gstk1* in zebrafish using the CRISPR/Cas9 system (Aida et al., 2015). Recent technological advances in genome editing have enabled us to make KO zebrafish for any gene of interest and validated the characterization of KO zebrafish at F0 (Kotani et al., 2015; Sasagawa et al., 2016b). As shown in Supplementary Figure S2, multiple heteroduplexes (red line) above the homoduplex (black arrowhead) were observed when PCR was performed using genomic DNA from zebrafish injected with crRNA for *gstk1*. These results suggest that the gstk1 crRNA efficiently edited the *gstk1* gene, resulting in *gstk1*-KO. We then performed qPCR to examine the effect of *gstk1*-KO on the expression of *acta1a*, *acta1b*, *vmhc*, *actr1*, *ctgfa*, and *postnb*, the zebrafish homologs of *ACTA2*, *ACTA1*, *MYH7*, *ACTR1B*, *CTGF*, and *POSTN*, respectively. As shown in **Figure 4**, *acta1a* expression was significantly increased in *gstk1*-KO zebrafish compared with the control zebrafish line. The expression of *acta1b* and *vmhc* was slightly increased in the *gstk1*-KO zebrafish (*p* = 0.05 and 0.06, respectively). By contrast, there were no significant differences in *actr1*, *ctgfa*, and *postnb* expression between the *gstk1*-KO and control zebrafish. These results suggest that *GSTK1* downregulation may increase the expression of sarcomeric genes, including *ACTA2*, *ACTA1*, and *MYH7*.

### *gstk1* Knockout Decreases the Cardiac End Diastolic and Systolic Volume in Zebrafish

**FIGURE 5:**
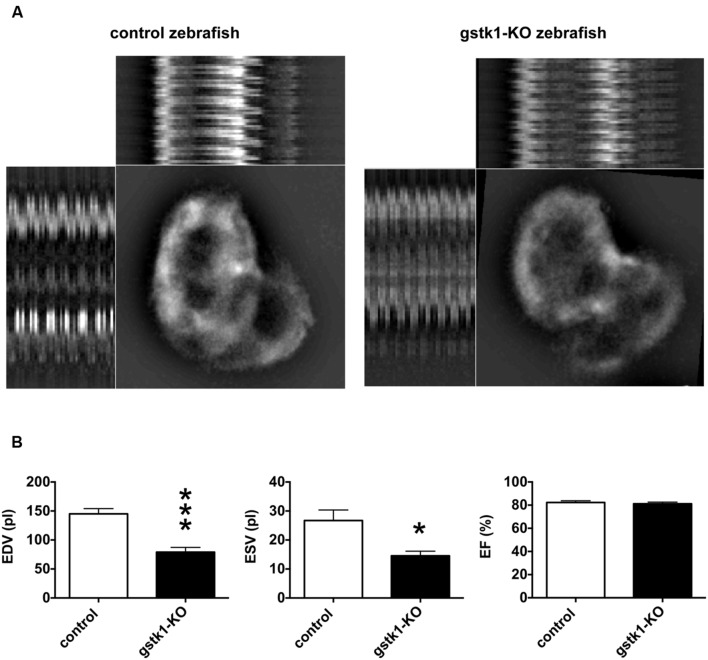
***gstk1* knockout decreases the cardiac end diastolic and systolic volumes in zebrafish. (A)** *In vivo* imaging of the hearts of control and *gstk1*-knockout (KO) Tg (myl7:mRFP) zebrafish at 5 dpf. Zebrafish were placed on slides on their backs, and the heart was imaged under a fluorescence microscope at 100 frames/s for 10 s. Image stack projections and the M-mode imaging of ventricles are shown. Bar, 100 μm. **(B)** Quantitative analysis of the *in vivo* imaging of zebrafish heart. The end diastolic volume (EDV) of *gstk1*-KO zebrafish was significantly smaller than that of control zebrafish, whereas the end systolic volume (ESV) and ejection fraction (EF) were not significantly different. *N* = 13 and 7 for the control and *gstk1*-KO groups, respectively. *p < 0.05, ***p < 0.001 vs. control group.

We next investigated the cardiac function of *gstk1*-KO zebrafish expressing mRFP in cardiomyocytes. The animals were examined by fluorescence microscopy and images were captured for measurement of diastolic and systolic diameters and calculation of EDV, ESV, and EF. We observed that the EDV in *gstk1*-KO zebrafish was significantly smaller than that of control zebrafish (**Figure 5**). The ESV was also significantly reduced in *gstk1*-KO zebrafish compared with that in control zebrafish, albeit to a lesser extent than the EDV. These results suggest that downregulation of *GSTK1* and subsequent upregulation of sarcomeric genes may be a common pathophysiological mechanism in HCM.

## Discussion

### *MYH7*, *CTGF*, *POSTN*, *RTN4*, and *GSTK1* are Dysregulated in HCM

We demonstrated that expression of *Myh7*, *Ctgf*, *Postn*, and *Rtn4* is significantly increased and expression of *Gstk1* is significantly decreased in five mouse HCM models. These results suggest that the five genes may be robust biomarkers of disease and/or involved in the pathogenesis of HCM.

*MYH7*, which encodes cardiac muscle β myosin heavy chain, is the major sarcomeric protein. Expression of *MYH7* is increased in the hearts of HCM patients compared with healthy subjects (Kontaraki et al., 2007). Our bioinformatic analysis revealed that SRF may increase the expression of *MYH7*, consistent with previous reports (Nelson et al., 2005). These findings suggest that *MYH7* upregulation, possibly through SRF activation, may be a common pathophysiological pathway in HCM.

CTGF and POSTN are extracellular matrix proteins regulated by TGFβ signaling (Grotendorst, 1997; Horiuchi et al., 1999). Previous work has shown that *Ctgf* and *Postn* expression is increased in mouse models of HCM caused by Myh6-R403Q (Teekakirikul et al., 2010; Tsoutsman et al., 2013) and by KO of *Mybpc3* (Judge et al., 2015). It was suggested that the source of *Ctgf* and *Postn* may be cardiac fibroblasts (Teekakirikul et al., 2010; Seidman and Seidman, 2011). TGFβ signaling is activated in various HCM models, and inhibition of TGFβ reduces fibrosis and limits hypertrophy remodeling (Teekakirikul et al., 2010; Seidman and Seidman, 2011). Thus, increased expression of *CTGF* and *POSTN*, potentially via TGFβ signaling, may underlie the fibrosis and collagen deposition associated with HCM of various etiologies.

RTN4, also called Nogo-A, is enriched in endoplasmic reticulum and known to be increased in genetic models of dilated cardiomyopathy and in end-stage heart failure in humans (Bullard et al., 2008). Knockdown of Nogo-A inhibited hypoxia/reoxygenation-induced activation of mitochondrial-dependent apoptosis in cardiomyocytes (Sarkey et al., 2011). These findings suggest that increased expression of *RTN4* may also be associated with HCM through impairment of mitochondria.

GSTK1 is a member of the κ class of glutathione S-transferases and is localized in mitochondria and peroxisomes (Morel et al., 2004; Raza, 2011). Proteomic analysis revealed that Gstk1 expression is decreased in HCM caused by pressure overload (Aubert et al., 2016) and diabetic cardiomyopathy (Das et al., 2015). *Gstk1* expression is increased by peroxisome proliferator-activated receptor α (PPARα) agonists (Knight et al., 2008). Given that PPARα signaling is impaired in HCM (Taegtmeyer et al., 2004), these observations suggest that *GSTK1* expression may be reduced, possibly via inhibition of PPARα, in HCM caused by various mechanisms.

### Reduction of *GSTK1* Expression is Associated with HCM

In this study, we demonstrated that KO of *gstk1* in zebrafish increased the expression of *ACTA2*, *ACTA1*, and *MYH7* homologs and decreased the EDV and, to a lesser extent, the ESV, consistent with a possible causative role for *GSTK1* downregulation in HCM.

GSTK1 is localized in mitochondria and peroxisomes (Raza, 2011), which are the important sites for lipid metabolism and oxygen consumption in cardiomyocytes (Colasante et al., 2015). Knockdown of *gstk1* and *gstk2* in *Caenorhabditis elegans* impairs oxygen consumption and lipid metabolism (Petit et al., 2009). In contrast, overexpression of *GSTK1* reduces lipid peroxidation in peroxisomes (Wang et al., 2013). Peroxisome-derived oxidative stress can cause mitochondrial damage (Wang et al., 2013). Oxidative stress, lipid peroxidation, and mitochondrial dysfunction have been associated with HCM (Vakrou and Abraham, 2014); the suppression of each function mitigated HCM (Liu et al., 2012). These findings suggest that down-regulation of *GSTK1* may cause HCM through increased oxidative stress, lipid peroxidation, and mitochondrial dysfunction. Increased oxidative stress activates SRF (Jin et al., 2011), suggesting that down-regulation of *GSTK1* may activate SRF through increasing oxidative stress, resulting in increased SRF-target genes such as *ACTA2*, *ACTA1*, and *MYH7* (Miano et al., 2007). However, the magnitude of the increase in the homologs of *ACTA1* and *MYH7* in gstk1-KO zebrafish was not strong. Moreover, the expression of the homologs of *CTGF* and *POSTN* was significantly increased in the five mouse HCM models but not in *gstk1*-KO zebrafish. These results suggest that down-regulation of *GSTK1* and other signaling pathways may synergistically cause HCM in mammals. Further studies using various models such as *Gstk1*-KO mice and induced pluripotent stem cells derived from HCM patients will be necessary to elucidate the detailed mechanisms by which downregulation of *GSTK1* may cause HCM.

## Author Contributions

YN conceived the study, performed the bioinformatics analyses, and wrote the manuscript. SS performed the experiments using the *gstk1*-KO zebrafish. SO performed the quantitative analysis of *in vivo* imaging of zebrafish hearts. SM, YA, MY, KK, and RK provided assistance with experiments. RO and MI wrote the manuscript. TT conceived the study and wrote the manuscript.

## Conflict of Interest Statement

The authors declare that the research was conducted in the absence of any commercial or financial relationships that could be construed as a potential conflict of interest.

## Abbreviations

ACTA1: actin alpha 1
ACTA2: actin alpha 2
CRISPR: clustered regularly interspaced short palindromic repeats
crRNA: CRISPR RNA
CTGF: connective tissue growth factor
dpf: days-post-fertilization
EDV: end diastolic volume
ESV: end systolic volume
EF: ejection fraction
DEG: differentially expressed gene
FA: Friedreich’s ataxia
FXN: frataxin
GEO: Gene Expression Omnibus
GSTK1: glutathione S-transferase kappa 1
HCM: hypertrophic cardiomyopathy
KO: knockout
mRFP: monomeric red fluorescent protein
MYH6: myosin heavy chain 6
MYH7: myosin heavy chain 7
MYL7: myosin light chain 7
PLN: phospholamban
POSTN: periostin
qPCR: quantitative polymerase chain reaction
RTN4: reticulon 4
SRF: serum response factor
TAC: transverse aortic constriction
TF: transcription factor
TPM1: tropomyosin 1.
