# Supplementary figures and images for: Downregulation of GSTK1 Is a Common Mechanism Underlying Hypertrophic Cardiomyopathy
Source: Front Pharmacol. 2016 Jun 14;7:162. doi: 10.3389/fphar.2016.00162 (PMC4905960; doi:10.3389/fphar.2016.00162)

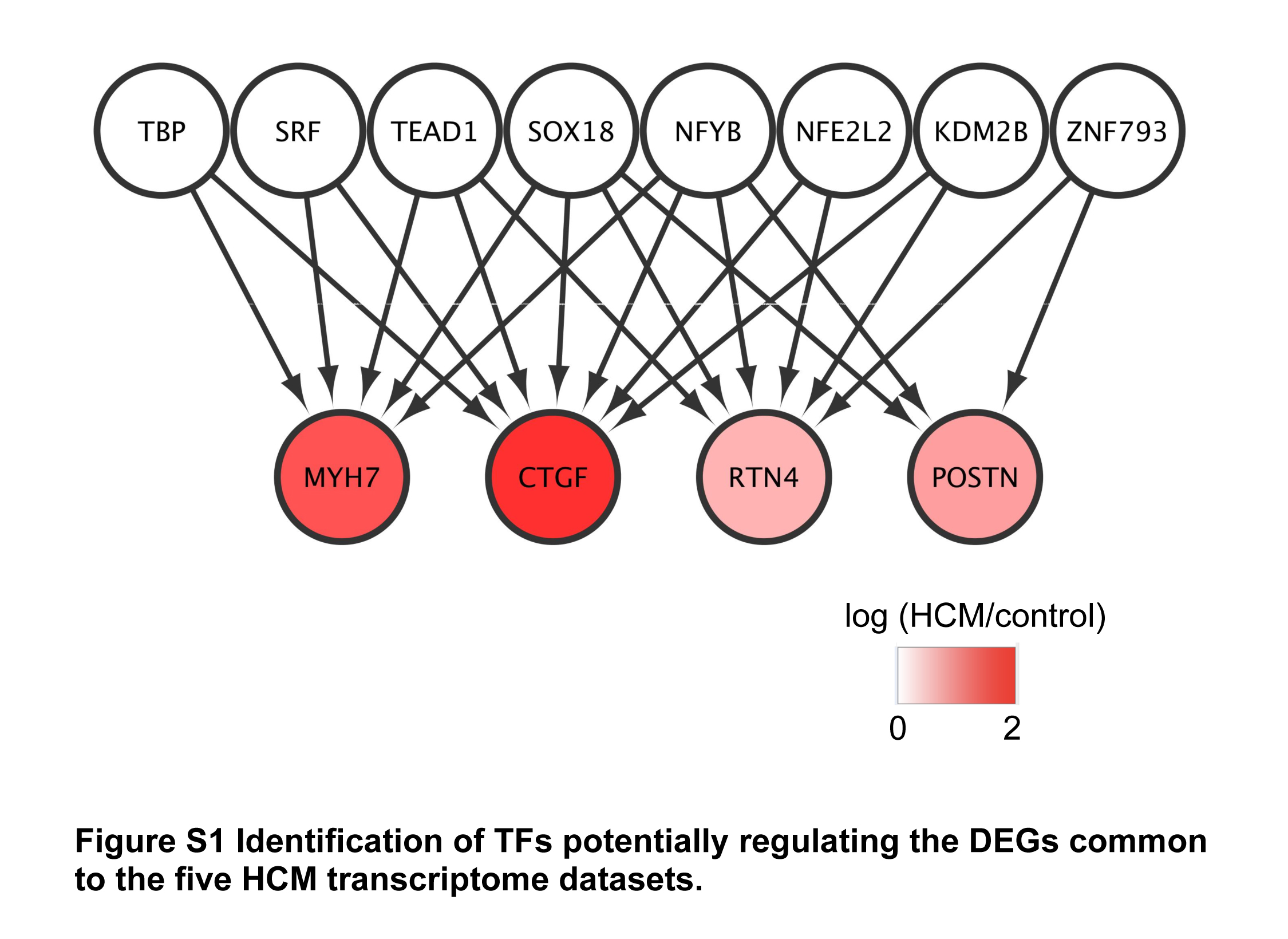

Supplement: Supplementary file 1 [file Image_1.JPG]

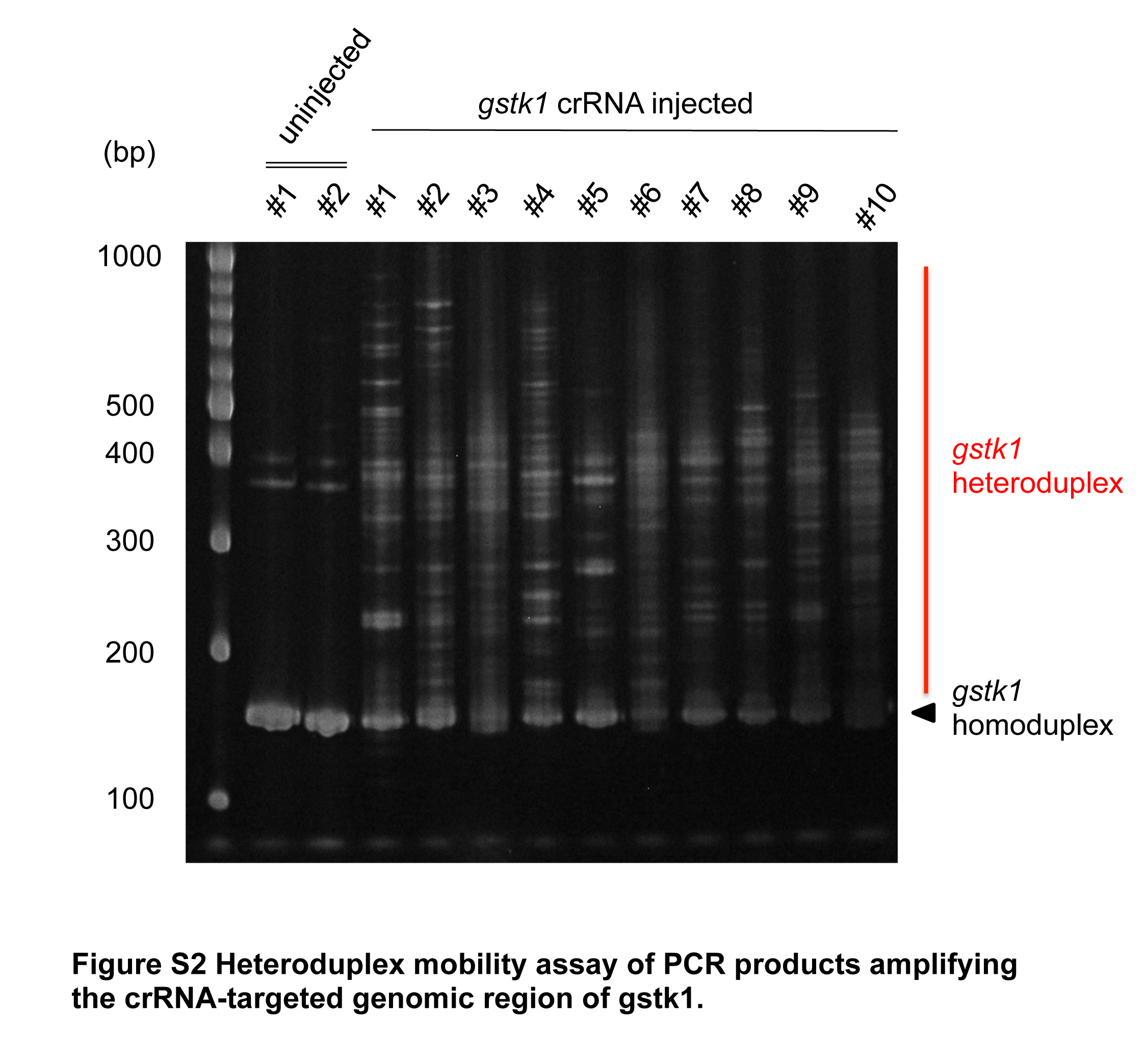

Supplement: Supplementary file 2 [file Image_2.JPG]
